# Assessment of knowledge, attitude and practice and associated factors towards palliative care among nurses working in selected hospitals, Addis Ababa, Ethiopia

**DOI:** 10.1186/1472-684X-13-6

**Published:** 2014-03-04

**Authors:** Hiwot Kassa, Rajalakshmi Murugan, Fissiha Zewdu, Mignote Hailu, Desalegn Woldeyohannes

**Affiliations:** 1 Department of Nursing, College of Medicine and Health Sciences, University of Gondar, Gondar, Ethiopia; 2 Department of Nursing, College of Health Sciences, Addis Ababa University, Addis Ababa, Ethiopia; 3 Department of Public Health, School of Medicine and Health Sciences, Addis Ababa Science and Technology University, Addis Ababa, Ethiopia

**Keywords:** Addis Ababa, Attitude, Knowledge, Nurses, Palliative care and practice

## Abstract

**Background:**

To provide quality care at the end of life or for chronically sick patients, nurses must have good knowledge, attitude and practice about palliative care (PC). In Ethiopia PC is new and very little is known about the type of services offered and the readiness of nurses to provide PC.

**Methods:**

A cross sectional quantitative study design was carried out using 341 nurses working in selected hospitals in Addis Ababa from January 2012 to May 2012. Systematic random sampling was the method employed to select two governmental and two non-governmental hospitals. The researchers used triangulation in their study method making use of: Frommelt’s Attitude Toward Care of the Dying (FATCOD) Scale, Palliative Care Quiz for Nursing (PCQN) and practice questions. This led to enhanced validity of the data. EPI-INFO and SPSS software statistical packages were applied for data entry and analysis.

**Result:**

Of the total 365 nurses selected, a response rate of 341 (94.2%) were registered. Out of the total study participants, 104 (30.5%) had good knowledge and 259 (76%) had favorable attitude towards PC. Medical and surgical wards as well as training on PC were positively associated with knowledge of nurses. Institution, individuals’ level of education, working in medical ward and the training they took part on PC were also significantly associated with the attitude the nurses had. Nurses working in Hayat Hospital (nongovernmental) had a 71.5% chance of having unfavorable attitude towards PC than those working in Black Lion Hospital (governmental). Regarding their knowledge aspect of practice, the majority of the respondents 260 (76.2%) had poor implementation, and nearly half of the respondents had reported that the diagnosis of patients was usually performed at the terminal stage. In line with this, spiritual and medical conditions were highly taken into consideration while dealing with terminally ill patients.

**Conclusion:**

The nurses had poor knowledge and knowledge aspect of practice, but their attitude towards PC was favorable. Recommendations are that due attention should be given towards PC by the national health policy and needs to be incorporated in the national curriculum of nurse education.

## Background

“Palliative care (PC) is an approach that improves the quality of life of patients and their families facing the problem associated with life-threatening illness through the prevention and relief of suffering by means of early identification, impeccable assessment and treatment of pain and other problems like physical, psychosocial and spiritual” [1]. By 2020, the World Health Organization (WHO) estimates that non-communicable diseases (NCDs) will be as prevalent as communicable diseases, which have been the main cause of high morbidity and mortality in sub-Saharan Africa. Despite the importance of PC in while managing NCDs, its limited development across Africa indicates many patients have not received formal PC services [2]. However, the development of PC through effective, low cost approaches is a feasible alternative response to the urgent needs of the sick and improve their quality of life [3]. It is a major undertaking for health systems worldwide to deliver appropriate PC. There are serious deficits in this field in many countries, and the need for PC will further increase as a result of demographic development with increasing numbers of older people with incurable chronic disease and multiple morbidities [4].

Based on a review of PC development in 47 African countries, only four countries were reported to have hospice and PC services are approaching some measure of integration, were reported [5]. These challenges are exacerbated by poor health and social care infrastructures as well as limited health financing in many African countries. In addition, there is lack of understanding of what PC is and what its benefits are [5]. Undeniably, Ethiopia is attempting to move forward in its policy development process for establishing PC [6]. However, it is estimated that only 15% of the over 80 million people are able to access healthcare, hence most people who are suffering from cancers, human immunodeficent virus/acquired immunodeficient syndrome (HIV/AIDS), and other chronic illnesses are often diagnosed late stage. This creates a huge burden of suffering for the Ethiopian people with extremely limited access to pain medications and other PC interventions. There are no governmental programs in Ethiopia for PC, but there are only three non-governmental organizations, which provide support for chronically ill patients at their residence; consequently, meeting the PC needs of cancer and HIV/AIDS patients has not received considerable public health priority [6]. However, Hospice Ethiopia, a local nongovernmental organization, started in 2004 in the country and is trying to address this problem through care for patients with chronic illnesses, HIV and cancer [7].

For low resource countries, hospice and PC services are often provided voluntarily by health professionals, such as nurses, in addition to their regular full-time job [8]. Nurses are by far the largest healthcare providers in almost every country. However, despite this type of support for PC, nursing has lagged behind other disciplines for not incorporating PC into the nursing education curricula. This underdeveloped educational foundation has difficulties in defining the role of nurses in PC [9]. For such reasons further efforts are needed to push the PC initiative across the health care continuum [10]. Improving PC will require significant investment in research, facilitation of clinical care and to furnish appropriate education to health care providers. Inadequate research hampers our understanding of patient and care-giving mechanisms, as well as the identification of effective solutions [11]. Therefore, the value of PC to nurses who deliver majority of care to chronically ill patients is unquestionable, and there is a need to support and educate nurses for the provision of high quality palliative and end-of-life care. Hence, the first step in developing a strategy to support and educate nurses about PC is to assess their current knowledge, attitudes and practice as there is limited research on PC with nurses. To this end, the aim of this is study was to assess the knowledge, skills, attitudes and associated factors with PC in nurses working in selected hospitals in Addis Ababa, Ethiopia.

## Methods

### Study design, area and period

A cross-sectional study was conducted with nurses at Governmental and Non-Governmental hospitals in Addis Ababa, Ethiopia from January 2012 to May 2012.

### Subjects

This study was confined to nurses working in Governmental and Non-Governmental hospitals. Of the total number of institutions found in the city four were selected randomly. The nurses serving in outpatient departments and wards of the selected hospitals were recruited to participate in the study regardless of their years of service. However, nurses working in the central sterilization supply department, operating room, neonatal unit and delivery rooms were excluded.

### Sample size

The sample size was determined using single population proportion formula, since the total sample size was less than 10,000, we applied a correction factor; thus, the initial sample size was 221. However, as the sample selection passed through two stages (multistage sampling), we used a design effect of 1.5 and add 10% for non response to reach a total sample size of 365.

### Sampling technique

Hospitals found in Addis Ababa were stratified into Governmental and Non-governmental. Then two hospitals from each were selected using simple random sampling. In these institutions, the number of subjects assigned in wards were 191, 96, 50 and 28 for Black Lion, St Paul, Betel and Hayat; respectively, keeping proportionality into consideration.

### Data collection instrument and technique

A self administered English questionnaire was used for data collection. The attitude scale was adopted from Frommelt Attitude Toward Care of the Dying (FATCOD) and modified so as to make it fit to Ethiopian context. The knowledge questions were adopted from the Palliative Care Quiz for Nursing (PCQN) which was also modified according to the prevailing context of health institutions in Ethiopia. The practice questions were also adopted from different related studies. However, the tool was validated in English and not translated to local language.

The data collection instrument included four sections. Section one: A socio demographic variables include (age, gender, institution, ward, level of education, work experience, experience of caring terminally ill and PC training). Section two: before pretest the attitude was measured through the original FATCOD questionnaire which consists of 30 items however, after pre test the questionnaires were reduced to 24 items because some of the questions were difficult to understand and increase bulkiness of the questioner. The tool has a 5 point Likert scale. This was used to represent people's attitudes to a topic scored on 5 point scale, i.e. 1 (Strongly Disagree), 2 (Disagree), 3 (Uncertain), 4 (Agree) to 5 (Strongly Agree). Twelve of the items were worded positively, and twelve were worded negatively. Thus, possible score range was 0 to 120. A higher score indicates a more positive attitude toward PC. The third section included knowledge questions which came from the Palliative Care Quiz for Nursing (PCQN) using questions with Yes, No, or Don’t know answers. A high score indicates better knowledge [12]. The last section had 12 practical questions which the researchers constructed from guidelines and various literatures related to PC practice.

A Pre-test was conducted on 10% (34) of the nurses in one Governmental hospital in Addis Ababa. This helped us to verify the validity and reliability issues. The questionnaire was revised based on the findings of the pilot test.

### Data collection and quality control

Data collection was conducted by four graduate nurses. Two were from the Governmental and two from the Nongovernmental hospitals. Data collectors received a half day training on issues concerning the questionnaire (on the objective of the study, the how of approaching the participants, how to administer and collect the questionnaires timely was done). To successfully accomplish this research, the preparation of appropriate instruments as well as human resources, like assistants, were undeniably vital. Consequently, the questionnaire was revised before data collectors were disseminated to the actual data collection sites. Confidentiality of the study participants were kept during distribution and data collection periods. Above all, ethics, coding and entry were maintained throughout the process.

### Statistical analysis

The data was first entered into EPI-INFO version 3.5.1 then exported to SPSS version 16 statistical software packages. Chi-square and logistic regression were computed in order to assess statistical association and to see the level of significance, respectively.

### Operational definition

Favorable attitude = ≥ 50 of the total score of (FATCOD) Scale.

Unfavorable attitude = < 50% of the total score of (FATCOD) Scale.

Good knowledge = ≥75% of total score of the Palliative Care Quiz for Nursing (PCQN) scale

Poor knowledge = < 75% of total score of the PCQN scale

Knowledge aspect of practice = the nurse must have a knowledge on PC regarding application of practice.

Good knowledge aspect of practice = ≥75% of total knowledge aspect of practice questions.

Poor knowledge aspect of practice = < 75% of total knowledge aspect of practice questions.

### Ethical consideration

Ethical clearance was obtained from the Institutional Review Board (IRB) of Addis Ababa University (AAU)-College of Health Sciences, Department of Nursing and Midwifery, which also facilitated an official letter written to the selected hospitals and to the Addis Ababa City Administration Health Bureau to get their permission and cooperation for the study. Approvals were also obtained from participating hospitals. Verbal consent was obtained from each participants, and participants’ anonymity and confidentiality was kept. The respondents had had the right not to participate in or withdraw from the study at any stage.

## Result

### Socio-demographic characteristics of nurses

The total number of participants was 365 and the response rate was 341 (93.4%). The number of participants by hospital were from Black lion 176 (51.6%), St. Paul 88 (25.6%), Betel 46 (13.5%) and Hayat 31 (9.1%). Among 341 nurses who completed the questionnaire the majority of the participants 225 (66%) were female and the mean age of the respondents was 30.98 years ± 8.75 SD (range from 20 to 60). The respondents were working in pediatrics 94 (27.6%), outpatient 94 (27.6%), medical 84 (24.4%), surgical 93 (27.3%) and oncology 10 (2.9%) wards (see Table 1).

**Table 1:** Socio-demographic characteristics of nurses at selected hospitals in Addis Ababa, May 2012

**Characteristics**	**Frequency**	**Percentage**
	No (341)	% (100)
Institution		
Black Lion	176	51.6
St Paul	88	25.8
Hayat	31	9.1
Betel	46	13.5
Age		
20-30 years	216	63.3
31-40 years	67	19.6
41-50 years	44	12.9
>50 years	14	4.1
Level of education		
Diploma	170	49.9
Degree	171	50.1
Ward		
Medical	84	24.4
Surgical	93	27.3
Oncology	10	2.9
Pediatric	94	27.6
Out patient	66	17.6
Working experience		
Less than 5 years	182	53.4
5-10 years	70	20.5
11-15 years	36	10.6
>20 years	53	15.5
Experience in caring terminally ill patient		
Daily	186	54.5
Once per week	70	20.5
Once per month	27	7.9
Few times per year	33	9.7
Never	25	7.3
Training		
Yes	74	21.7
No	267	78.3
How long		
1-2 weeks	54	15.8
6 month	20	5.9
Never	267	78.3

### Nurses’ knowledge towards PC

Nearly 70% of the respondents knew the definition of PC and 82.2% agreed that PC is being given when patient’s conditions are deteriorating. Similarly 82% of nurses responded that addiction is noticed as the major health problem when morphine is used in long term. Forty four percent of the subjects agreed that accumulation of losses render burn out for those who work in PC. Of the total respondents 69.5%, 66% and 71.6% agreed that adjuvant therapies are important in pain management, that the patients right not to resuscitate (DNR) should be respected, and that terminally ill patients should be encouraged to have hope, respectively. Again, out of the total study participants only 104 (30.5%) had good knowledge towards PC (see Table 2).

**Table 2:** Distributions of nurses’ knowledge towards palliative care at selected hospitals in Addis Ababa, May 2012

**No**	**Characteristic**	**Yes N (%)**	**No N (%)**	**Don’t Know N (%)**
1	Do you know the definition palliative care?	237 (69.5)	30 (8.8)	74 (21.7)
2	Palliative care is only appropriate in situations of a downhill trajectory or deterioration in conditions.	280 (82.1)	45 (13.2)	16 (4.7)
3	The extent of the disease determines the method of pain treatment.	290 (85)	43 (12.6)	8 (2.3)
4	Adjuvant therapies are important in managing pain.	237 (69.5)	30 (8.8)	74 (21.7)
5	Drug addiction is a major problem when morphine is used on a long-term basis for the management of pain.	280 (82.1)	45 (13.2)	16 (4.7)
6	The provisions of palliative care require emotional detachment.	142 (41.6)	160 (46.9)	39 (11.4)
7	During the terminal stages of an illness, drugs that can cause respiratory depression are appropriate for the treatment of severe dyspnea.	81 (23.8)	181 (53.1)	79 (23.2)
8	The philosophy of palliative care is compatible with that of aggressive treatment.	112 (32.8)	156 (45.7)	73 (21.4)
9	The use of placebos is appropriate in the treatment of some types of pain.	204 (59.8)	61 (17.9)	76 (22.3)
10	Meperidine (Demerol®) is not an effective analgesic for the control of chronic pain.	97 (28.4)	117 (34.3)	127 (37.2)
11	The accumulation of losses renders burnout Inevitable for those who work in palliative care.	150 (44)	76 (22.3)	115 (33.7)
12	Manifestations of chronic pain are different from those of acute pain.	297 (87.1)	34 (10)	10 (2.9)
13	Terminally ill patients have the right to choose “Do not resuscitate” (DNR).	225 (66)	86 (25)	30 (8.8)
14	Terminally ill patients should be encouraged to have hope against all odds.	244 (71.6)	67 (19.6)	30 (8.8)

### Distribution of nurse’s attitude according to degree of agreement towards items of FATCOD

Nearly half of the participant nurses strongly agreed that PC was given only for dying patients. More than half of the respondents strongly disagreed to withdrawing their involvement with patients who are at the verge of death. One hundred thirty four (39.3%) and 43 (12.6%) agreed and strongly agreed; respectively, that it is possible for nurses to help patients prepare for death using various psychological mechanisms. On the other hand, over half of the nurses 174 (51%) agreed that family should be concerned about helping their dying member; likewise, nearly half of the respondents 170 (49.9) agreed that patients and family should be in charge of making decisions about patients end of life care. In contrast 103 (30.2%) and 83 (34.3%) of respondents felt uncomfortable talking about death with a dying patient and they usually refused to be assigned to give care for dying people, respectively. In general, more than three quarters of the respondent (76%) had favorable attitude towards PC (see Table 3).

**Table 3:** Distribution of nurses attitude according to their degree of agreement toward items of FATCOD at selected hospitals in Addis Ababa, May 2012

**No**	**Statement**	**SD (%)**	**D (%)**	**U (%)**	**A (%)**	**SA (%)**
1	Palliative care is given only for dying patient.	168 (49.)	103 (30.2)	13 (3.8)	35 (10.3)	17 (5)
2	As a patient nears death; the nurse should withdraw from his/her involvement with the patient.	227 (66.6	72 (21.1)	4 (1.2)	30 (8.8)	8 (2.3)
3	Giving nursing care to the chronically sick patient is a worthwhile learning experience.	38 (11.1)	41 (12)	37 (109)	139 (40.8)	86 (25.2)
4	It is beneficial for the chronically sick person to verbalize his/her feelings.	27 (7.9)	22 (6.5)	28 (8.2)	154 (45.2)	110 (33)
5	Family members who stay close to a dying person often interfere with a professionals' job with the patient.	49 (13.5)	95 (27.9)	44 (12.9)	123 (36.1)	33 (9.7)
6	The length of time required to give nursing care to a dying person would frustrate me.	97 (28.4)	108 (31.7)	50 (14.7)	68 (19.9)	17 (5)
7	Families should be concerned about helping their dying member make the best of his/her remaining life.	30 (8.8)	20 (5.9)	19 (5.6)	174 (51)	98 (28.7)
8	Family should maintain as normal an environment as possible for their dying member.	21 (6.2)	35 (10.3)	27 (7.9)	187 (54.8)	71 (20.8)
9	The nurse should not be the one to talk about death with the dying person.	74 (21.7)	86 (25.2)	35 (10.3)	103 (30.2)	43 (12.6)
10	The family should be involved in the physical care of the dying person.	43 (12.6)	46 (13.5)	36 (10.6)	133 (39)	83 (24.3)
11	It is difficult to form a close relationship with the family of a dying member.	65 (19.1)	102 (29.9)	55 (16.1)	85 (24.9)	34 (10)
12	There are times when death is welcomed by the dying person.	30 (8.8)	37 (10.9)	19 (5.6)	141 (41.3)	114 (33.4)
13	Nursing care for the patient's family should continue throughout the period of grief and bereavement.	41 (12.0)	59 (17.3)	42 (123)	139 (40.8)	60 (17.6)
14	The dying person and his/her family should be the in-charge decision makers.	25 (7.3)	38 (11.1)	36 (10.6)	170 (49.9)	72 (21.1)
15	Addiction to pain relieving medication should not be a nursing concern when dealing with a dying person.	111 (32.6)	99 (29)	23 (6.7)	74 (21.7)	34 (10)
16	Nursing care should extend to the family of the dying person.	59 (17.3)	66 (19.4)	33 (9.7)	128 (37.5)	47 (13.8)
17	When a patient asks, “Nurse am I dying?'I think it is best to change the Subject to something cheerful.	43 (12.6)	73 (21.4)	56 (16.4)	123 (36.1)	46 (13.5)
18	I am afraid to become friends with chronically sick and dying patients.	110 (32.5)	131 (38.4)	22 (6.5)	61 (17.9)	17 (5)
19	I would be uncomfortable if I entered the room of a terminally ill person and found him/her crying.	93 (27.3)	102 (29.9)	24 (6.5)	93 (27.3)	29 (8.5)
20	I would be uncomfortable talking about impending death with the dying Person.	65 (19.1)	91 (26.7)	36 (10.6)	103 (30.2)	46 (13.5)
21	It is possible for nurses to help patients prepare for death.	68 (19.9)	58 (17)	38 (10.6)	134 (39.3)	43 (12.6)
22	Death is not the worst thing that can happen to a person.	91 (26.7)	101 (29.6)	36 (10.6)	69 (20.2)	44 (12.9)
23	I would feel like running away when the person actually died.	132 (38.7)	101 (29.6)	29 (8.5)	55 (16.1)	24 (7)
24	I would not want to be assigned to care for a dying person.	121 (35.5)	103 (30.2)	34 (10)	58 (17)	25 (7.3)

SD: strongly disagree, D:disagree, U: uncertain, A: agree, SA: strongly agree.

### Knowledge aspect of practice of nurses towards PC

Two thirds (76.2%) of the respondents had poor knowledge towards PC. Only 54.2% of them initiating PC discussions with patients during diagnosis while 167 (49%) of nurses inform terminally ill patients about their diagnosis. Regarding decision making, 205 (60.1%) of the respondents reported obtaining patients’opinions and 111 (32.6%) involved the family in the decision making amongst the family. Regarding psychological issues of the patient, 285 (83.6%) of respondents reported hiding the truth from patients, 180 (52.8%) preferred to give emotional support, and 159 (46.6%) provided counseling to the patients. The majority (96.8%) of the respondents perceived terminally ill patients concern as need of treatment and 307 (90%) had attention seeking behavior, respectively. The commonly used medications for severe pain were paracetamol or ibuprofen 207 (60.7%), morphine108 (31.1%) and codeine 41 (12%) (see Table 4). Respondents mentioned that PC was provided most to patients with cancer (36.4) and HIV (28.4).

**Table 4:** Practice of nurses towards palliative care at Addis Ababa selected hospitals, May 2012

**No**	**Characteristics**	**Multiple response**	**Frequency**	**Percentage**
			**Yes n (%)**	**No n (%)**
1	Initiate palliative care discussion:	During diagnosis	186 (54.5)	155 (45)
		When the disease progress	128 (37.5)	213 (62.5)
		At the end of life	57 (16.7)	284 (83.3)
2	Do you inform terminally ill patient about their diagnosis?	Yes	167 (49)	174 (51)
		No	69 (20.2)	272 (79.8)
		Depending on family’s wish	96 (28.2)	245 (71.8)
		Inapplicable	11 (3.2)	330 (96.8)
3	Factors considered when dealing with terminally ill patient:	Spiritual	172 (50.4)	169 (49.6)
		Medical situation	149 (43.7)	192 (56.3)
		Cultural	69 (20.2)	272 (79.8)
		Psychological	124 (36.4)	217 (63.6)
4	Address spiritual issue:	Connect with spiritual counselor	177 (51.9)	164 (48.1)
		Listen with empathy	133 (39)	208 (61)
		Impose your own view	320 (94.7)	18 (5.3)
		Understand patient reaction	74 (21.7)	267 (78.3)
5	Cultural assessment during patient care should include:	Truth telling and decision making	148 (43.4)	193 (56.6)
		Preference regarding disclosure of information	55 (16.1)	286 (83.9)
		Dietary preference	65 (19.1)	276 (80.9)
		Language, family communication	158 (46.3)	183 (53.7)
		Perspective on death, suffering & grieving	42 (12.3)	299 (87.7)
6	Addressing psychological:	Emotional support	180 (52.8)	161 (47.2)
		Counseling the patient	159 (46.6)	182 (53.4)
		Hiding the truth	285 (83.6)	56 (16.4)
7	Whom do you involve in decision making?	Patient	205 (60.1)	136 (39.9)
		Family	111 (32.6)	230 (67.4)
		My own	27 (7.9)	314 (92.1)
		Other health professional	47 (13.8)	294 (86.2)
8	How do you perceived terminally ill patient concern or question?	Patient right	273 (80.1)	68 (19.9)
		Treat	336 (96.8)	11 (3.2)
		Doubting your professionalism	25 (7.3)	316 (92.7)
		Attention seeking behavior	307 (90)	34 (10.0)
9	Communication to the family of terminally ill patient depends on:	Family’s ability to assimilate	230 (67.7)	111 (32.6)
		Their involvement in decision making	206 (60.4)	135 (39.6)
		Your willingness to disclose information	36 (10.6)	305 (89.4)
10	Commonly use medication in your practice for severe pain?	Paracetamol/Ibuprofin	207 (60.7)	134 (39.3)
		Codeine	41 (12)	300 (88)
		Morphine	108 (31.7)	233 (68.3)
11	How do you assess patient pain?	Grade with face	201 (58.8)	140 (41.1)
		Intensity	122 (35.8)	219 (64.2)
		Location	34 (10)	307 (90)
		Quality	39 (11.4)	302 (88.5)

### Association between socio-demographic variables and nurses attitude towards PC

Institution, level of education, ward, and training had significant association with attitude of nurses. There were no statistically significant relationships between age, gender, work experience, experience in caring for terminally ill patients, and duration of training with nurses’ attitude towards PC. In addition, our findings revealed that nurses working in the Hayat Hospital had 28.5% [AOR = 0.285; CI 0.121-0.669; p = 0.009] unfavorable attitude compared to the nurses at the Black Lion Hospital. Similarly, nurses who had a bachelors degree had revealed twice exceeding positive attitude [AOR = 2.415; CI 1.383- 4.218; p = 0.003] compared to those who held a diploma. On the other hand, nurses working in a medical ward were threefold better in their positive attitudes than those who work in outpatient department [AOR = 3.44; CI 1.410- 8.398; p = 0.002]. Lastly, nurses trained on PC had more favorable attitude towards PC compared to nurse who did not take PC training [AOR = 2.218; CI 1.039, 4.731; P = 0.025] (see Table 5).

**Table 5:** The association of socio-demographic characteristics and attitude of nurses towards palliative care at selected hospitals in Addis Ababa, May 2012

**Characteristic**		**Attitude**		**P value (x**^**2**^**)**	**COR 95(CI)**	**AOR 95(CI)**
		**Good n(%)**	**Poor n(%)**			
Institution	Black lion	137 (40.2)	39 (11.4)	0.009* (11.477)	1.00	1.00
	St. Paul	71 (20.8)	17 (5)		1.189 (0.628,2.25)	1.395 (0.703,2.767)
	Hayat	16 (4.7)	15 (4.4)		0.304* (0.138,0.668)	0.285* (0.121,0.669)
	Betel	35 (10.3)	11 (3.2)		0.906 (0.421,1.947)	0.96 (0.416,2.15)
Age	20-30 years	162 (47.5)	54 (15.8)	0.556 (2.08)		
	31-40 years	52 (15.2)	15 (4.4)			
	41-50 years	36 (10.6)	8 (2.3)			
	> 50 years	9 (2.6)	5 (1.5)			
Gender	Male	90 (26.4)	26 (7.6)	0.709 (0.139)		
	Female	169 (49.6)	56 (16.4)			
	Total	259 (76)	82 (24)			
Level of education	Diploma	117 (34.3)	53 (15.5)	0.003* (8.627)	1.00	
	Degree	142 (41.6)	29 (8.5)		2.218* (1.326,3.711)	2.415* (1.383,4.218)
	Total	259 (76)	82 (24)			
Ward	Medical	84 (24.6)	10 (2.9)	0.002* (14.547)	3.6* (1.528,8.483)	3.44* (1.41,8.398)
	Surgical	70 (20.5)	23 (6.7)		1.304 (0.63,2.695)	1.153 (0.535,2.484)
	Pediatric	63 (18.5)	31 (9.1)		0.871 (0.433,1.754)	0.297 (0.34,1.522)
	Out patient	42 (12.3)	18 (5.3)		1.00	1.00
Working Experience	Less than5 years	137 (40.2)	45 (13.2)	0.677 (2.320)		
	5-10 years	55 (16.1)	15 (4.4)			
	11-15 years	28 (8.2)	8 (2.3)			
	> 20 years	39 (11.4)	14 (4.1)			
Experience in caring terminally ill patient	Daily	143 (41.9)	43 (12.6)	0.911 (0.537)		
	Once per week	50 (14.7)	20 (5.9)			
	Once permonth	23 (6.7)	4 (1.2)			
	Few times per year	24 (7)	9 (2.6)			
	Never	19 (5.6)	6 (1.8)			
Training	Yes	64 (18.8)	10 (2.9)	0.025* (8.627)	2.365* (1.151,4.851)	2.218* (1.039,4.731)
	No	195 (57.2)	72 (21.1)		1.00	
How long	1-2 weeks	45 (13.2)	9 (2.6)	0.728 (0.121)		
	1 month	18 (5.3)	2 (0.6)			

* Significant P≤ 0.05 level.

### Association between socio-demographic variables and nurses knowledge towards PC

Only ward setting and training had a significant association with level of knowledge; however, institution, age, gender, level of education, work experience, experience of caring for terminally ill patient and duration of training did not. Working in medical [AOR = 2.751; 95% CI 1.223 -6.188; P = 0.003] and surgical [AOR = 3.445 CI 1.479-4.399; P = 0.003] wards affected PC positively. On the otherhand, nurses who had training on PC had approximately three times greater knowledge [AOR = 2.551; CI 1.479-4.399; P = 0.001] than those who had no such training (see Table 6).

**Table 6:** The association of socio-demographic characteristics and knowledge of nurses towards PC at selected hospitals in Addis Ababa, May 2012

**Characteristic**		**Knowledge**		**P value (x**^**2**^**)**	**COR 95(CI)**	**AOR 95(CI)**
		**Good n(%)**	**Poor n(%)**			
Institution	Black lion	55 (16.1)	121 (35.5)	0.717 (1.353)		
	St. Paul	27 (7.9)	61 (17.9)			
	Hayat	11 (3.2)	20 (5.9)			
	Betel	11 (3.2)	35 (10.3)			
Age	20-30 years	62 (18.2)	154 (45.2)	0.781 (1.084)		
	31-40 years	23 (6.7)	44 (12.9)			
	41-50 years	15 (4.4)	29 (8.5)			
	> 50 years	4 (1.2)	10 (2.7)			
Gender	Male	34 (10)	82 (24)	0.732 (0.48)		
	Female	70 (20.5)	155 (45.5)			
Level of education	Diploma	48 (14.1)	122 (35.8)	0.365 (0.620)		
	Degree	56 (16.4)	115 (33.7)			
Ward	Medical	34 (10)	60 (17.6)	0.003* (13.784)	3.833* (1.275,6.29)	2.751* (1.223,6.18)
	Surgical	38 (20.5)	55 (16.1)		3.445* (1.560,7.64)	3.445* (1.537,7..72)
	Pediatric	22 (6.5)	72 (21.1)		1.528 (0.666,3.504)	1.515 (0.653,3.515)
	Out patient	10 (2.9)	50 (14.7)		1	1
Working Experience	>5 years	48 (14.1)	134 (39.3)	0.306 (3.617)		
	5-10 years	25 (7.3)	45 (12.9)			
	11-15 years	14 (4.1)	22 (6.5)			
	> 20 years	17 (5)	36 (10.6)			
Experience in caring terminally ill patient	Daily	60 (17.6)	126 (37.0)	0.764 (1.847)		
	Once/week	17 (5)	53 (15.5)			
	Once/month	9 (2.6)	18 (5.3)			
	Few times per year	11 (3.2.)	22 (6.5.)			
	Never	7 (2.1)	18 (5.3)			
Training	Yes	35 (11.1)	39 (11.4)	0.001*	2.575* (1.512,4.38)	3.023* (1.750,5.22)
	No	69 (20.2)	198 (58.1)		1	1
How long	1-2 weeks	23 (31.1)	31 (41.9)	0.285 (1.145)		
	1 month	12 (16.2)	8 (10.8)			

* Significant P≤ 0.05 level.

## Discussion

The result of this study showed that the majority of nurses had poor knowledge about PC. The possible reason for this might be that only a few nurses ‘have been trained on PC. Studies in Australia and New Zealand showed that training on PC was the most frequently nominated professional need among nurses [13,14]. This result was consistent with other studies done in Florida, California and India [10,11,15]. Researchers have also documented the serious deficiencies in undergraduate nursing education and in nursing knowledge and attitudes related to end-of-life care [16]. The findings from this study had also confirmed the strong association of the type of wards and training on attitude towards PC. Though some studies showed that age, past and present experience with death, education regarding end of life care and year of clinical experience had significant influence on ones attitude towards PC [17]. The result of this study showed that those factors were not significantly associated with participants ‘care-giving attitude.

The findings of the present study on the level of knowledge among nurses contradict with other studies [9,18]. Furthermore, this result also disagreed with studies conducted in Florida and Lebanon [19,20]. This might be due to the fact that PC education was not incorporated into either diploma or degree curricula.

In this study the majority, 76%, of nurses had favorable attitude towards PC, which is also evident in other studies [9,13,18,21,22]. Those factors that were significantly associated with nurse’s attitude in this study might be due to the fact that in Ethiopia, trainings are given most of the time for staff in governmental hospitals. The study also showed that nurses working in the medical ward had strong association compared to outpatient department. The possible reason for this might be chronic illnesses patients are mostly admitted to the medical ward and, thus, nurses who worked in the medical ward had daily contact with those patients, and may have developed favorable attitude towards PC.

Nurses who had a higher education degree (BSc.) in this study had had two fold increasing favorable attitude compared to diploma graduate nurses. The reason for this might be bachelorette nurses are able to understand the FATCOD scale in a better way than that of diploma graduates. The finding is consistent with Egypt and South Africa studies [21,23].

The negative association of age, gender, work experience, experience of caring terminally ill patients, and duration of training with nurses’ knowledge might be due to PC is a novice discipline in Ethiopia. This finding contradicts with a South Africa study [23], United State and Iranian studies examine the effect palliative education has on nurses’ attitude and the result showed significant increase in nurses’ attitude compared to the time before training [17,24]. New England and Denmark studies also support this finding [22,25].

Almost two thirds of the respondents in this study agreed that PC is worthwhile learning experience. The finding is consistent with studies done in Denmark and Egypt [21,22]. In this study 36.5% of the nurses were uncomfortable about talking about death with dying patients and it was lower than the result from Egypt 43.7% and higher than from South African 24% [21,23]. This difference might be because of cultural differences related to delivering bad news or talking about death in front of the patient, difficult topics to discuss in Ethiopian culture and can potentially have psychological impact on patients and their families. Twenty four percent of the nurses did not want to be assigned to care for a dying patient, which is slightly higher compared to other studies [17], [20-23]. Nearly half of the respondents in the present study believed to change the subject to something cheerful when a patient asks a nurse, “Am I dying?” This finding contradicts with other studies [21,23]. Moreover, research results in Denmark and Norway asserted that the majority of nurses viewed withholding information from patients, or lying to patient about their diagnosis and prognosis, as unethical [25]. The reason for hiding the truth from the patient might be related with fear of nurses to confront the dying patient, and probably because they might feel that they are not be competent enough since majority of them didn’t take PC training and they don’t know how to handle the condition. However, different literatures support that it is important to tell patients they are dying so that they have opportunities to process the implications of dying, to reconcile with loved ones, to leave legacies or explore the meaning of their lives [26]. Concerning the responsibility of nurses about drug addiction, in the present study 61.5% of the respondents believed that drug addiction should be a concern of a nurse when dealing with dying patients, which was higher compared to other findings [21,23].

Regarding knowledge aspect practice of nurses, majority 76.2% had good practice which contradicts with the result from New Heaven [27]. Poor knowledge aspect of practice in this finding could be related with respondents’ poor knowledge towards PC and it might also be due to the study subjects who had less than five years of work experience since experience might affect the practice. Forty nine percent of participated nurses inform terminally ill patients about their diagnosis which was higher compared to studies done in Lebanon, United States, and England [19,20,27] and lower from study in Norway [25]. Since diagnosis of patients used to be expressed by physicians, nurses may not dare to disclose to patients in the case of Ethiopia. The finding of this study reveal that half of the respondents consider spiritual and 43.7% prefer medical treatments other than cultural and psychological beliefs when treating terminally ill patients. This finding is concurrent to the study in Lebanon study [19]. This could be due to Ethiopians great value and concern for religion.

The majority 83.6% of the nurses in this study addressed psychological issues of the patient by hiding the truth. In contrary, study done in Norway reported that majority of nurses viewed that lying to the patients about their diagnosis and prognosis as unethical [25]. Nearly two thirds (60.7%) of the respondents’ use paracetamol, or ibuprofen for chronic pain management this might be a result of unavailability of opioid analgesics and/or poor awareness about chronic pain management. Similarly, a study done in Malawi, health workers required access to pain medication and knowledge of oral morphine in order to provide appropriate patients care [28]. This is important because unrelieved pain has a serious effect on the quality of life, interfering with sleep, daily activity, enjoyment of life and social interaction [29].

### Limitations of the study

Shortage of similar studies carried out in Ethiopia, Africa and in other parts of the world makes the comparison and discussion difficult. Lack of a standard tool for practice and unavailability of PC unit in the hospitals involved were challenges of this study to assess the real practice of nurses on PC.

## Conclusion

The literatures suggest that nurses can have a prominent role in end-of-life care. Hence it is important to assess nurses’ knowledge, attitude and practice to help them handle such cases. The result of this study suggested that the majority of respondents that have had favorable attitude but poor knowledge and practice towards PC. Similarly, ward and training on PC were significantly associated with knowledge; institution, level of education, ward and training, on the other hand, were found to be statistically significant with the attitude of nurses towards PC. In conclusion much should be done to assist nurses perform their duties based on the knowledge they grasp in various trainings, workshops, formal or informal education. The Departments of Nursing in higher education institutions in Ethiopia should also incorporate courses related to PC issues so as to strengthen their graduates’ level of understanding.

## Abbreviations

AIDS: Acquired immunodeficiency syndrome; EOL: End of life; FATCOD: Frommelt attitude toward care of the dying; HIV: Human immunodeficiency virus; NCD: Non Communicable disease; NGO: Non-governmental organization; PC: Palliative care; PCQN: Palliative care quiz for nursing; WHO: World Health Organization.

## Competing interests

The authors declare that they have no competing interests.

## Authors’ contributions

HK: initiation of the study, design, implementation, analysis and write-up as well as prepared the manuscript for publication. RM: initiation of the study, design, analysis and writing. FZ: Design and write-up. MH: design and implementation. DW: design, analysis and write-up as well as prepared the manuscript for publication. All authors read and approved the final manuscript.

## Pre-publication history

The pre-publication history for this paper can be accessed here:

http://www.biomedcentral.com/1472-684X/13/6/prepub
